# Current Treatment of Juvenile Myelomonocytic Leukemia

**DOI:** 10.3390/jcm10143084

**Published:** 2021-07-13

**Authors:** Christina Mayerhofer, Charlotte M. Niemeyer, Christian Flotho

**Affiliations:** 1 Division of Pediatric Hematology and Oncology, Department of Pediatrics and Adolescent Medicine, Medical Center, Faculty of Medicine, University of Freiburg, 79106 Freiburg, Germany; christina.mayerhofer@uniklinik-freiburg.de (C.M.); charlotte.niemeyer@uniklinik-freiburg.de (C.M.N.); 2 German Cancer Consortium (DKTK), 79106 Freiburg, Germany

**Keywords:** juvenile myelomonocytic leukemia, RAS signaling, hematopoietic stem cell transplantation, 5-azacitidine, myelodysplastic/myeloproliferative disorders, targeted therapy

## Abstract

Juvenile myelomonocytic leukemia (JMML) is a rare pediatric leukemia characterized by mutations in five canonical RAS pathway genes. The diagnosis is made by typical clinical and hematological findings associated with a compatible mutation. Although this is sufficient for clinical decision-making in most JMML cases, more in-depth analysis can include DNA methylation class and panel sequencing analysis for secondary mutations. *NRAS*-initiated JMML is heterogeneous and adequate management ranges from watchful waiting to allogeneic hematopoietic stem cell transplantation (HSCT). Upfront azacitidine in *KRAS* patients can achieve long-term remissions without HSCT; if HSCT is required, a less toxic preparative regimen is recommended. Germline *CBL* patients often experience spontaneous resolution of the leukemia or exhibit stable mixed chimerism after HSCT. JMML driven by *PTPN11* or *NF1* is often rapidly progressive, requires swift HSCT and may benefit from pretransplant therapy with azacitidine. Because graft-versus-leukemia alloimmunity is central to cure high risk patients, the immunosuppressive regimen should be discontinued early after HSCT.

## 1. Introduction

JMML is a pediatric leukemia with shared features of myelodysplastic and myeloproliferative neoplasms, usually manifesting during early childhood with leukocytosis, thrombocytopenia, pronounced monocytosis, splenomegaly, immature precursors on peripheral blood (PB) smear, and bone marrow (BM) blast count below 20% [1,2,3]. Its clinical and hematological picture, as well as natural history and outcome, are remarkably diverse [4]. The common molecular denominator of JMML is the deregulation of the intracellular Ras signal transduction pathway, caused in >90% of cases by mutations in one (or, rarely, more than one) of five primordial genes (*PTPN11*, *NRAS*, *KRAS*, *NF1*, or *CBL*) [5]. For most patients, allogeneic hematopoietic stem cell transplantation (HSCT) is the only curative treatment option, in contrast to a smaller percentage of children who survive long-term without HSCT and eventually experience spontaneous clinical remissions [6,7]. Clinical and molecular risk factors were established to help predict the disease course and guide therapeutic decisions, including age at diagnosis, percentage of fetal hemoglobin (HbF), platelet count, and aberrant DNA methylation patterns [8,9]. In this article, we review the current knowledge of genetic and epigenetic properties of JMML and provide detailed recommendations for the clinical management of children diagnosed with this challenging disorder.

## 2. The Origin of JMML: The Ras Pathway

The Ras pathway is a sequence of kinases in the cell that serves as a chain of communication between extracellular mitogens and the cell nucleus [10]. External cytokine signals, relayed through receptor tyrosine kinases and intracellular adapter proteins, lead to guanosine exchange factor-mediated transformation of Ras proteins into their active guanosine triphosphate (GTP)-bound state (reviewed in more detail in [11,12]). The Ras signal is terminated by intrinsic Ras phosphatase activity, which converts Ras back to an inactive guanosine diphosphate (GDP)-bound configuration. An additional layer of regulation is provided by GTPase activating proteins (GAPs). Effects of Ras activation include the subsequent phosphorylation of Raf, Mek, and Erk kinases [13,14,15,16,17], activation of the mammalian target of rapamycin (mTOR) axis via phosphoinositide 3-kinase (PI3K) [18], and others [19]. Among nuclear targets are the transcription factors Jun and Fos [20].

Genetic mutations in specific Ras pathway components (*PTPN11*, *NRAS*, *KRAS*, *NF1*, or *CBL*), resulting in net hyperactivation of the Ras-GTP-GDP loop, are present in hematopoietic cells of >90% of children diagnosed with JMML [4,21,22,23,24,25,26]. These can be traced back to early myeloid stem/progenitor cell compartments [27,28,29], and they are found in patient cord blood samples [24], substantiating their role as initiating events and suggesting the inception of the leukemogenic sequence before birth [30].

Somatic mutations in exons 3 or 13 of the *PTPN11* gene are present in ~35% of JMML cases [22,31], resulting in a gain-of-function of the nonreceptor tyrosine phosphatase Shp2 [32]. Somatic mutations in *NRAS* or *KRAS* codons 12, 13, or 61, accounting for ~25% of JMML cases [4,25,33], freeze Ras in its active GTP-bound form by inhibition of GTPase activity or resistance to GAPs [4]. Somatic *PTPN11*, *NRAS*, and *KRAS* mutations occur in heterozygous form in JMML, indicating strong cell-transforming capacity already in monoallelic fashion.

Two congenital developmental disorders predispose to JMML: NF-1 and CBL syndrome [26,34,35,36]. Here, the germline of the patient carries a monoallelic loss-of-function mutation of the *NF1* or *CBL* gene, which may have been inherited or arisen de novo. JMML develops after somatic biallelic inactivation of the respective gene in hematopoietic progenitor cells, predominantly by mitotic gene recombination resulting in uniparental isodisomy [21,37]. *NF1* functions as a Ras-GAP and thus negatively regulates the Ras pathway [38,39]. Indicative features in children with JMML/NF-1 are the presence of ≥6 cutaneous café au lait spots and/or the family history; other characteristics of NF-1, such as neurofibromas, optic pathway gliomas, bone lesions and neurological abnormalities, usually manifest only later. Overall, 10–15% of JMML cases are driven by *NF1* [33,40]. CBL is a E3 ubiquitin ligase mediating the decay of receptor tyrosine kinases in the Ras pathway. Mutations targeting exons 8 or 9account for ~15% of JMML cases [26,33]. CBL syndrome, a Noonan-like rasopathy, has a wide phenotypic spectrum. Features include impaired growth, facial anomalies, developmental delay, cryptorchidism, autoimmune phenomena, and notably, neurovasculitis [26,37]. However, it is not rare for patients with JMML and *CBL* germline mutation to display no abnormalities at all [26,41,42].

Noonan syndrome (NS), the most common rasopathy with an incidence of 1 in 1000–2500 children [43], bears clinical similarities with Turner syndrome. Patients with NS exhibit a short statue, facial dysmorphism, congenital heart defects, skeletal defects, a webbed neck, mental retardation, and cryptorchidism. The genetic basis is a germline mutation in *PTPN11* (around 50% of NS cases), *SOS1*, *RAF1*, *KRAS*, *BRAF*, *NRAS* or other members of the RAS pathway [5,22,44,45]. Children with NS may experience a polyclonal myeloproliferative disorder (MPD) at a very young age, sometimes shortly after birth [4,46]. Although the condition is indistinguishable from JMML by clinical and hematological features, it has a self-limiting course in the vast majority of cases. Only a small fraction of children with NS/MPD progress to JMML, presumably after the acquisition of additional genetic changes [5,47]. Although the landscape of *PTPN11* mutations is not identical in JMML and NS/MPD [31], there is considerable overlap, and it is not well understood how the same mutation elicits a transient disorder when present in the germline and a fatal disorder when acquired somatically. Obviously, the occurrence of germline and somatic Ras pathway mutations in the same clinical context requires analysis of non-hematopoietic tissue (e.g., hair follicles or skin fibroblasts) to differentiate these conditions [9].

Systematic exome sequencing studies revealed that JMML is generally characterized by a paucity of somatic mutations in the neoplastic clone when compared to most other types of cancer [48]. However, subclonal secondary gene mutations can be found in up to half of the cases [23,24,48]. These mutations primarily target the *SETBP1*, *JAK3*, *SH2B3*, or *ASXL1* genes. Not infrequently, the secondary mutations affect the Ras pathway itself (“Ras double mutants”). In addition, a role for subclonal mutations in the Polycomb Repressive Complex 2 network was highlighted [23]. Several studies have linked the presence of secondary mutations with an aggressive clinical course or disease progression [23,49]. Furthermore, an association with an increased risk of recurrence after allogeneic HSCT was demonstrated [23,49].

Less than 10% of JMML cases are negative for the five canonical driver mutations. Rarely, these children harbor germline or somatic activating *RRAS* mutations [23,50]. Recently, a *CCDC88C-FLT3* fusion responsive to sorafenib was described in a pediatric patient with clinical features of JMML and monosomy 7 [51]. Other fusions detected in children with myeloproliferative disease include *ALK* [52,53], *ROS1* [52,53], *FIP1L1-RARA* [54], *HCMOGT-1-PDGFRB* [55], *NDEL1-PDGFRB* [56], and *NUP98-HOXA11* [57]. Although kinase fusion-positive cases without Ras pathway mutation may fulfill the clinical and diagnostic criteria of JMML, they likely represent a genetically distinct myeloproliferative neoplasm in childhood. When identified, these tyrosine kinase fusions offer an attractive target for personalized therapies [51,53].

## 3. Clinical and Hematological Features of JMML

JMML occurs in 1.2 children per million per year, accounting for 2% of pediatric hematopoietic malignancies [58]. One half of the children with JMML are diagnosed below the age of two years and two-thirds are male [59]. Clinical signs at diagnosis include non-specific symptoms such as infections, fatigue, or failure to thrive. Splenomegaly is noted in nearly all cases, often accompanied by hepatomegaly and lymphadenopathy. Pulmonary infiltration by leukemic cells manifests with dry cough, tachypnea and, radiologically, interstitial infiltrates [59,60]. Abdominal symptoms may arise in patients with intestinal infiltration [59]. Variable cutaneous features may be present, ranging from eczematous lesions to erythematous papules or nodules and/or petechiae [60]. In contrast to other pediatric leukemias, JMML does not usually invade the central nervous system. As a substantial proportion of JMML cases arise on the basis of an underlying predisposition syndrome, the clinician needs to examine the patient carefully, paying attention to growth, facial dysmorphism, congenital heart defects, skeletal anomalies, developmental status, and skin lesions such as café-au-lait macules or juvenile xanthogranulomas [45,61,62,63].

The PB smear typically shows mild to pronounced leukocytosis with monocytosis without a significantly increased number of blasts (median 2% myeloblasts) [60,64]. Immature precursor cells of the granulocytic lineage (myelocytes, metamyelocytes), immature monocytes and nucleated erythropoietic cells are found, giving the blood film examination a pivotal diagnostic role [59]. Platelet and erythrocyte counts are usually decreased, whereas the absolute monocyte count is increased to >1 × 10^9^ G/L in all but exceptional cases [1]. Bone marrow examination is necessary to exclude acute leukemia, but is per se insufficient to confirm a suspected diagnosis of JMML. BM findings in JMML include hypercellularity from myelomonocytic proliferation, reduction of megakaryocytes and moderate increase of blasts (<20% myeloblasts) [59].

The combination of young age, splenomegaly, skin lesions, appearance of myeloid and erythroid precursors in the PB, and/or elevated levels of HbF should prompt the pediatric oncologist to suspect JMML and initiate specific tests. First of all, this involves the molecular analysis of driver mutations in the *PTPN11*, *KRAS*, *NRAS*, and *CBL* genes, and a meticulous search for features of NF-1 including family history. Genetic analysis of *NF1* can be added, but it is laborious, and the interpretation of findings is not always straightforward. On cytogenetics, two-thirds of cases exhibit a normal karyotype. Monosomy 7 is the most frequent aberration [33,53,65], occurring in combination with *PTPN11* and *KRAS* mutations, but rarely with *NF1*, *NRAS*, or *CBL*. A traditional hallmark of clonogenic JMML cells is their hypersensitivity to GM-CSF in vitro [66,67]. However, laboratory tests of this feature are poorly standardized and not widely available. In the era of efficient mutational analysis, GM-CSF hypersensitivity has become largely dispensable, but may potentially be helpful in occasional cases without a canonical driver mutation. The direct antiglobulin test may come back positive due to autoantibodies, but this is usually not accompanied by clinical or laboratory hemolysis [59]. Similarly, increased levels of IgG, IgM and IgA can be observed [59]. It was suggested that flow cytometric analysis of STAT5 hyperphosphorylation after stimulation with GM-CSF may aid in distinguishing JMML from other conditions [68].

Bacterial [69] and viral (e.g., Epstein–Barr virus [70], cytomegalovirus [71], and herpesvirus 6 [72]) infections can mimic the clinical and laboratory findings of JMML in infants, including fever, splenomegaly, leukocytosis with monocytosis, hypersensitivity to GM-CSF, and STAT5 hyperphosphorylation. Genetic or non-leukemic hematological disorders, such as infantile malignant osteopetrosis, leukocyte adhesion deficiency, Wiskott–Aldrich syndrome, or Ras-related autoimmune lymphoproliferative disease (RALD), must also be differentiated from JMML [69,73,74]. The latter is a non-malignant, chronic condition induced by an apoptosis defect in lymphocytes [74,75]. RALD is characterized by monocytosis, lymphoproliferation and autoimmune phenomena. Blood leukocytes exhibit similar somatic *NRAS* and *KRAS* mutations as in JMML [74], but these patients do not require aggressive treatment. Two cases of JMML evolving from RALD were described in the literature [75,76], highlighting the need for close observation. Differentiation of both entities can be difficult in the absence of monosomy 7. Functional apoptosis assays might be helpful to diagnose RALD [75].

## 4. The Emerging Role of Epigenetics

The genetic subtypes discussed above account for the phenotypic diversity of JMML only incompletely. For example, long-identified prognostic parameters, such as age of the patient, sex, platelet count, or elevated levels of fetal hemoglobin [6,40,59], do not correspond to a specific Ras genotype. Further molecular factors related to the course of the disease were observed in JMML, including micro and long non-coding RNA expression [77,78], AML-like expression profile [79], secondary mutations [48,49] and alterations of the fetal hematopoietic regulator gene *LIN28B* [80]. In addition, the previous observation of epigenetic dysregulation during Ras-mediated transformation suggested aberrant DNA methylation as a potential disease modifier [81,82].

The first study examining the role DNA methylation changes in a large European series of 127 children with JMML revealed CpG island hypermethylation of a candidate gene set in up to half of the cases [81]. Importantly, CpG hypermethylation at diagnosis was an independent risk factor for poor overall survival (OS) and risk of relapse after HSCT [81]. The conclusions were largely confirmed in a Japanese study investigating a similar candidate gene set [83]. Both studies observed that hypermethylation in JMML affected a narrow subset of gene promoters, as opposed to broad random distribution across all genetic regions examined, suggesting that high-risk JMML is characterized by a CpG island hypermethylation phenotype, as discovered previously in other specific cancer types [82,83,84]. Several follow-up studies corroborated this concept at the candidate gene level [85,86,87].

Extending these findings to a genome-wide scope using array-based methods, study groups in Europe [33], Japan [53] and United States [65] analyzed independent JMML cohorts with the aim to establish a methylation based risk-stratification. Comparing the methylome patterns of 167 children with JMML, the European Working Group of Myelodysplastic Syndromes in Childhood (EWOG-MDS) discriminated 3 distinct methylation groups, again highlighting epigenetic dysregulation as a strong prognostic risk factor [33]. Factors associated with hypermethylation were repressed chromatin, Ras pathway double mutants and upregulation of methyltransferases DNMT1 and DNMT3B. This supported the emergence of DNA hypermethylation as a consequence of hyperactive Ras signaling [81,83]. Several associations between genetic driver mutation and DNA methylation pattern were noted. The group of patients with highest hypermethylation was dominated by somatic *PTPN11* mutation and older children, both known factors for inferior clinical course, whereas the low-methylation group was enriched for patients with NS/MPD, CBL cases, and young children with somatic *NRAS* mutations. The group with intermediate hypermethylation was characterized by somatic *KRAS* mutations and occurrence of monosomy 7 [33]. The Japanese cohort, consisting of 106 JMML cases, was split into two methylation groups [53]. In addition to known clinical risk factors, the high-methylation group involved cases with *NF1* or *PTPN11* mutations, secondary mutations, *LIN28B* overexpression and AML-like expression profile [53]. The North American study defined three similar methylation classes in 39 patients [65]. Interestingly, some JMML patients with good transplantation-free outcome and all patients with NS/MPD exhibited a DNA methylation signature closer to healthy controls than to other JMML cases [65,88], again underlining the significance of disrupted epigenetic control for the biology of JMML. The fact that all three methylome studies had used a comparable technical platform provided the unique opportunity for a comprehensive overarching meta-analysis. These collaborative efforts succeeded in developing and validating an international standard classifier of three different methylation categories matching those above and correlating with disease biology and outcome [88]. The prospective use of methylation analysis as a biomarker in JMML will aid in adapting treatment strategies, e.g., use of pretransplant therapy or low-intensity graft-versus-host disease (GVHD) prophylaxis, and support the generation of internationally comparable JMML study data.

## 5. Current Recommendations for the Management of JMML

**Figure 1:**
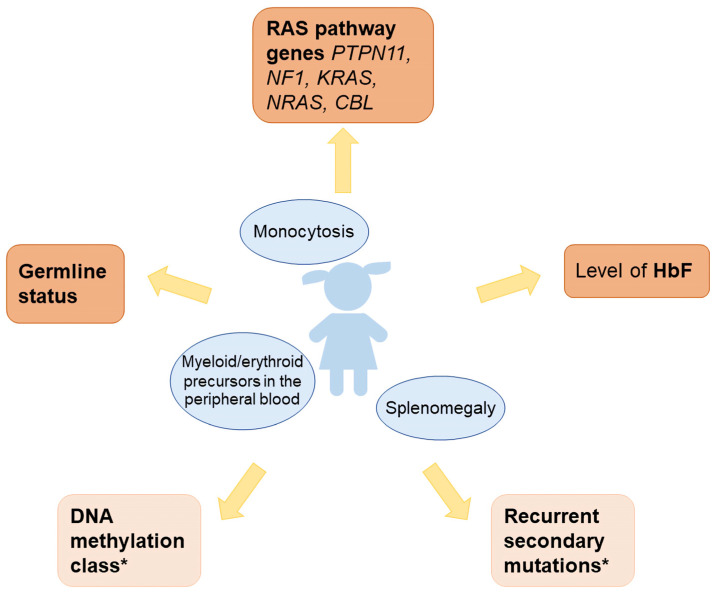
Diagnostic approach for children with JMML. * Helpful for clinical decision making, but not mandatory.

With extensive molecular diagnostic work-up of JMML established in major international study groups and large centers around the world, it has become evident that there can no longer be a uniform one-size-fits-all approach for this disorder (Table 1). The authors recommend that therapeutic decisions in a newly diagnosed case of JMML be based on the following diagnostic information (Figure 1):

- Level of fetal hemoglobin (measured in a blood sample taken prior to erythrocyte transfusion; levels far above the age-adjusted reference value are also meaningful if sampled after transfusion)
- Panel sequence analysis of the five primordial Ras pathway genes (*PTPN11*, *NF1*, *KRAS*, *NRAS*, *CBL*)
- Presence of the primordial mutation in non-hematopoietic tissue (indicating germline status)
- Where available, the following additional information will aid in clinical decision-making, though it is not indispensable for adequate management in most cases:
- DNA methylation class
- Panel sequence analysis of recurrent secondary mutations. The assessment should include *SETBP1*, *JAK3*, *RRAS*, *RRAS2*, and *ASXL1*; other targets are rare.

### 5.1. Somatic NRAS Mutation

The disease course in this group is remarkably heterogeneous. In older children with severe thrombocytopenia, increased HbF and high methylation class, a rapidly progressive course with a considerable risk of recurrence after HSCT is to be expected, likening the disorder to *PTPN11*- or *NF1*-driven JMML. On the other hand, a group of patients exist who are clinically well and have low HbF. Here, spontaneous clinical regression of the disease can occur in the long run without therapy. The search for an unrelated stem cell donor may be deferred in these cases. In between these two ends of the spectrum, the prospective identification of patients who benefit from watchful waiting is the real challenge. Factors suggesting surveillance without therapy include infant age, clinical status, age-appropriate levels of HbF, and low methylation class [41,89,90]. However, this must be balanced with the concern that delaying HSCT may compromise the outcome in some patients. EWOG-MDS data shows that the survival curve of JMML patients without HSCT keeps dropping during the first ten years and then plateaus at 25%.

It is very rare for a germline *NRAS* or *KRAS* mutation to be detected in a suspected JMML case. Most of these children have additional syndromic features of the rasopathy spectrum [91,92] or correspond in phenotype to Noonan syndrome [93]. Anecdotal observations contradict the paradigm that the canonical tumor-associated Ras mutations in codons 12, 13 or 61 are not tolerated in the germline; such cases are sometimes based on mosaicism [94,95]. Because of their rarity, no general recommendation can be given for the treatment of these highly individual cases.

### 5.2. Somatic KRAS Mutation

Children diagnosed with *KRAS*-JMML are typically very young, often infants. Concurrent monosomy 7 in the neoplastic clone is often observed (see below). Autoimmune phenomena (hyperimmunoglobulinemia, autoantibodies) should be searched for, and a diagnostic differentiation from RALD [74,96,97] should be kept in mind. The clinical presentation of *KRAS*-driven JMML tends to be aggressive, requiring rapid intervention. In the past, long-term survival without HSCT has not been reported for this group, but the picture is now changing with the introduction of azacitidine. *KRAS*-mutated JMML responds particularly well to low-dose azacitidine with long-lasting clinical and molecular remissions [98,99]. Regimens use 100 mg/m^2^/day on five consecutive days or 75 mg/m^2^/day on seven consecutive days, repeated every 28 days; due to instability, immediate intravenous or subcutaneous application of the cold reconstituted solution must be observed. Azacitidine has a favorable toxicity profile in children with JMML, mainly including lower-grade cytopenias, gastrointestinal discomfort, and infections [64,98]. It is variable how many cycles of azacitidine are necessary to achieve a response; between 6 and 9 cycles are usually administered. Frequently, the earliest sign of response to azacitidine is the improvement of thrombocytopenia. The spleen size diminishes after three to six cycles. Possibly, long-term cure with azacitidine alone will be achievable in *KRAS*-mutated JMML with a low risk profile.

### 5.3. Somatic PTPN11 Mutation

This is the numerically largest group that also carries the highest risk of rapid progression and early death. As no curative chemotherapy regimen is known, expeditious planning of allogeneic HSCT is mandatory. Within this group, risk factors for an unfavorable course are: age at diagnosis ≥2 years, significantly increased level of HbF, presence of secondary mutations, and/or high methylation class. In these cases, the aim should be to perform HSCT within a period of 3 months after diagnosis (recommendations for implementation see below). Low-dose azacitidine is the preferable option for upfront therapy until HSCT in non-high risk cases, with the goal to achieve a more favorable biological status of the leukemia. In many affected children, this treatment leads to a clinical partial remission or at least sufficient disease control until HSCT [64,98,99,100] However, high-risk cases carry the potential of immediate progression under azacitidine and may therefore be better off with more instant cytoreduction using 6-mercaptopurine (50 mg/m^2^/day, to be adjusted according to clinical course) and/or cytarabine (40 mg/m^2^/day × 5 days) [7]. If a patient presents in a critically ill condition that requires rapid reduction of tumor burden, the use of intensive chemotherapy with cytarabine (2 g/m^2^/day × 5 days) and fludarabine (30 mg/m^2^/day × 5 days) may also be considered [7]. However, this involves a substantial risk of organ toxicity and life-threatening infections. Removal of the spleen, which is often grossly enlarged, can be justified in individual cases to control respiratory impairment. A systematic beneficial effect of splenectomy on the further course of the disease has not been proven [40,101]. It is not yet clear whether treatment with azacitidine before HSCT also improves the long-term prognosis in this patient group. A recent non-randomized prospective study using a matched historical cohort as control group supports this assumption [98]. Until more precise evidence is available, the authors emphasize that treatment with azacitidine must not delay urgent HSCT in patients with *PTPN11* mutation.

### 5.4. Germline Mutation in PTPN11

Germline mutations in *PTPN11* cause Noonan syndrome, a condition of the rasopathy spectrum predisposing to a myeloproliferative disorder imitating JMML. For this reason, genetic diagnostics in suspected cases of JMML must always be carried out in both hematopoietic and non-hematopoietic material. Cultivated fibroblasts from a skin biopsy are ideal; hair follicles are less invasive, but more challenging to analyze. An oral mucous membrane swab has a high probability of contamination with hematopoietic cells, even if it is macroscopically not bloody, and should therefore only be scored if the result is negative.

In cases of suspected JMML in very young patients or with clinical evidence of Noonan syndrome, the diagnostic test for *PTPN11* should not just encompass the hotspot exons 3 and 13 because Noonan syndrome mutations may also be found in exons 4 or 8. The spectrum of somatic *PTPN11* mutations in nonsyndromic JMML and germline *PTPN11* mutations in Noonan-associated MPD overlaps to a great extent but not completely [31].

The distinction between non-syndromic JMML and Noonan-associated MPD is important, as the latter is usually self-limiting. However, these patients must be monitored carefully, as there can be relevant clinical compromise from cell infiltrates, making it necessary to begin cytoreductive therapy. In individual cases, a clonal development towards a bona fide neoplastic disease is possible [47,102,103].

### 5.5. Neurofibromatosis Type 1

If not already recognized in the patient, the syndrome can usually be diagnosed clinically and/or through family history at the time of onset of JMML. In younger children, only café-au-lait spots but not the other typical signs of neurofibromatosis may be present. Six or more café-au-lait spots as stipulated in the NIH criteria are noted in the majority of children, but in exceptional cases there may be none or fewer. Genetic analysis of *NF1* usually confirms the clinical diagnosis in children with JMML/NF-1 [104]. The typical finding is an *NF1*-inactivating heterozygous variation in the germline of the patient that arose de novo (~50% of cases) or was inherited. These lesions are often, but not always, deletions or truncating missense mutations reported as recurrent aberrations in the NF-1 literature. In addition, the neoplastic clone exhibits somatic loss of heterozygosity at the *NF1* locus or an independent second *NF1* mutation, leading to biallelic *NF1* inactivation [21,104,105]. The judgment is more difficult if no clinical signs of neurofibromatosis are present and the genetic findings correspond to the above paradigm only incompletely (for example, in case of monoallelic lesions, variants of unclear significance, no germline findings or low allelic frequency). In such cases, a myeloid disorder with a secondary *NF1* lesion, but driven by an unrelated event, may be present. With careful work-up, however, such dilemmas are rare.

JMML on the basis of NF-1 manifests more frequently at an older age than the other groups and typically does not involve a drastically reduced platelet count. Some children initially show little clinical impairment. However, long-term survival without HSCT has not yet been observed in this group, so that proceeding to transplant and inception of therapy with azacitidine is advisable as in patients with somatic *PTPN11* mutation. Consistent with this recommendation, JMML/NF-1 cases almost always have an intermediate or high methylation profile [88].

### 5.6. CBL Mutation

The typical configuration of *CBL* mutations in JMML is a heterozygous missense point mutation in *CBL* exons 8 or 9 in the germline, accompanied by uniparental isodisomy of the 11q chromosome arm as a somatic event in hematopoietic cells, leading to loss of heterozygosity [26,36,37]. Many, but not all, children show syndromic rasopathy features, such as facial dysmorphia and growth retardation. A particular phenomenon in this patient group is the frequent occurrence of autoimmunity and vasculitis. Some children with *CBL*-JMML have massive organ enlargement and may require splenectomy for symptom relief. Most patients do not require swift HSCT but can be managed with watchful waiting; many of these experience spontaneous resolution of the myeloproliferation. The homozygous *CBL*-mutant status in hematopoietic cells may persist until adulthood even in the absence of hematologic abnormalities [106]. Patients who undergo transplant often revert to stable mixed chimerism with sufficient disease control [6,26,36]. It is still unclear if the allograft also prevents the later development of symptoms related to autoimmune vasculitis. A recent report highlighted the role of somatic-only *CBL* inactivation in five patients with a clinical course that required HSCT [42], again illustrating the need for proper germline analysis in the diagnostic evaluation of JMML.

### 5.7. None of the Above

Suspected cases of JMML with negative panel sequencing for all five primordial genes and no clinical evidence of NF-1 are called “quintuple-negative” or “all-negative”. In a third of these cases, in-depth multimodal genetic analysis uncovered a driving role of the *NF1* gene in the absence of clinical NF-1 features [104]. In other cases, RNA sequencing identified activating fusions involving *ALK*, *ROS1*, or *FLT3* [51,53]. Some authors argue in favor of diagnosing such myeloproliferative disorders as JMML due to the indistinguishable clinical and hematologic presentation. Excluding non-neoplastic causes of myelomonocytic proliferation, a maximum of 5–10% of suspected JMML cases remain genetically unexplained. It is advisable to refer these patients to an extended rasopathy work-up, especially if additional syndromic stigmata are present.

### 5.8. Monosomy 7

The significance of monosomy 7 for the biology of JMML is unclear, and there seems to be no association with clinical features or relevance for outcome [40]. Interestingly, monosomy 7 is observed more frequently in European compared to Japanese patients with JMML [33]. In a large international series, all patients with monosomy 7 and intermediate methylation class carried *KRAS* mutations, in contrast to an association between monosomy 7 and *PTPN11* or *NF1* in the high methylation group, and the absence of monosomy 7 in patients with low methylation pattern [88]. The mechanistic connection between this particular chromosomal lesion and aberrant DNA methylation patterns is not understood. Overall, it is likely that the presence of monosomy 7 plays a supportive role in JMML rather than being an independent pathogenetic factor [107]. This concept is also supported by the observation of secondary monosomy 7 in a watch-and-wait patient with Noonan syndrome and neonatal myeloproliferative disorder [102].

### 5.9. Allogeneic HSCT

Busulfan-based myeloablative conditioning regimens are commonly chosen and achieve 55–73% OS with a moderate 10–15% rate of transplant-related mortality but significant probability of leukemia relapse in the order of 25–35% [7,40,101,108,109,110,111,112]. The EWOG-MDS currently recommends a three-alkylator regimen consisting of busulfan (0.8–1.2 mg/kg/dose given 4 doses per day, day 7 to day 4), cyclophosphamide (60 mg/kg/d, day 3 to day 2), and melphalan (125–140 mg/m^2^/d on day 1) [40]. In an attempt to reduce toxicity, a recent prospective randomized trial compared busulfan, cyclophosphamide, and melphalan with busulfan and fludarabine alone but terminated early due to excessive disease recurrence in the latter arm [113]. Matched sibling donors (MSD) or matched/1-antigen-disparate unrelated donors (MUD) are considered the most suitable stem cell sources [40]. Matched cord blood units are a viable alternative, especially for smaller patients [114,115,116,117]. Although haploidentical relatives are readily available for urgent transplant and highly motivated, this should still be viewed as an approach with limited experience [101,112,118]. A recent study from China in 47 JMML patients suggested a lower relapse incidence in mismatched/haploidentical donor transplants compared to matched donors with similar rates of acute/chronic graft-versus-host disease and non-relapse mortality [101].

The North American group noted better post-HSCT outcome of patients with JMML who experienced molecular response to pretransplant chemotherapy [119], similar to other pediatric leukemias [120,121]. However, a limitation was that only a minority of patients responded to chemotherapy, conceivably those with favorable disease biology. Biomarkers predicting response to chemotherapy are lacking. Therefore, it cannot be generalized that pretransplant chemotherapy benefits survival in JMML, and the risk of unwarranted organ damage remains a concern [122].

Beside the leukemia biology factors discussed above, the way the transplant procedure is handled significantly influences the risk of relapse. It is likely that it is not so much the conditioning regimen but rather the establishment of a graft-versus-leukemia effect that is decisive for the success of allogeneic HSCT in JMML [101,109,110,111]. For this reason, EWOG-MDS recommends keeping immunosuppressive therapy with cyclosporine A at low levels (trough levels around 80 µg/L) and tapering early (from day +40 in the absence of grade II-IV GVHD). It is advisable to determine the recipient-donor chimerism at very close intervals (up to weekly in high-risk patients), as the reappearance of even small autologous cell populations mandates immediate withdrawal of the immunosuppressive therapy [123,124,125].

Age at diagnosis ≥2 years, *NF1* or somatic *PTPN11* mutation, and high DNA methylation define a patient group whose risk of JMML recurrence after HSCT is even higher than 50%, bringing up the question of post-transplant prophylaxis. On the basis of favorable data for other myeloid neoplasms [126,127,128,129,130,131] and in the absence of better alternatives, the authors consider it appropriate to recommend azacitidine (started as soon as safe and tolerable after engraftment; 32 mg/m^2^/day for five consecutive days, every 28 days) plus donor lymphocyte infusions (started after 3 cycles of azacitidine and 4 weeks after discontinuation of immunosuppressive prophylaxis, CD3^+^ cell dose 1–5 × 10^6^/kg, repeated every 8 weeks with increasing cell dose up to 1–5 × 10^7^/kg). However, we emphasize that there are no systematic data for this approach in JMML.

### 5.10. Experimental Agents and Targeted Therapy

Despite the prominent role of the Ras/MAPK network, attempts to target this complex signal cascade have shown limited therapeutic benefit in JMML [10,132]. The Children’s Oncology Group is currently recruiting patients for a phase II trial to examine the safety and efficacy of oral trametinib, a MEK1/2 inhibitor, in refractory or relapsed JMML (NCT03190915). In vitro data from induced pluripotent stem cell lines suggests mutation-specific sensitivity to kinase inhibition, with a preferential sensitivity of *PTPN11*-driven JMML to trametinib [133]. BCL2 inhibition gave impressive results when combined with azacitidine in elderly AML patients [134,135] and early results argue for a benefit in pediatric patients with advanced MDS/AML [136,137]. With an upregulation of the macrophage immune checkpoint CD47 in myeloid malignancies, ongoing preclinical and clinical trials test CD47-directed agents in MDS/AML, with encouraging efficacy results in combination with azacitidine [138,139,140].

## Figures and Tables

**Table 1:** Management of JMML according to driver mutation. HSCT, hematopoietic stem cell transplantation; GVHD, graft-versus-host disease; DLI, donor lymphocyte infusions; NF-1, neurofibromatosis 1.

Ras Pathway Mutation	Frequency in JMML	Features	DNA Methylation Profile	Recommendations for Treatment
Somatic *NRAS*	10–15%	Diverse	Mostly low, occasional IM or HM	HSCT for many, careful selection of candidates for watch-and-wait
Somatic *KRAS*	10–15%	Frequent monosomy 7, autoimmune phenomena	Intermediate or low	Azacitidine and/or HSCT
Somatic *PTPN11*	35%	Compromised clinical status at diagnosis, highest risk of unfavorable outcome	Mostly high	Swift HSCT (+pretransplant azacitidine) with low intensity GVHD prophylaxis, in absence of GVHD early withdrawal of prophylaxis, consider azacitidine plus DLI posttransplant
Germline *NF1*	10–15%	Café-au-lait spots, possibly positive family history, older age at diagnosis, less severe thrombocytopenia	High or intermediate	Swift HSCT (+pretransplant azacitidine) with low intensity GVHD prophylaxis, in absence of GVHD early withdrawal of prophylaxis
Germline *CBL*	15%	Syndromic rasopathy features, autoimmunity and vasculitis	Low	Watch-and-wait. HSCT if disease progresses, patients after HSCT often revert to stable mixed chimerism
All negative	5–10%	Rarely activating kinase fusions in RNA sequencing	Low or intermediate	Differentiate non-neoplastic disease, perform extended work-up for rasopathies. Most patients require HSCT
